# Visual Function in Eyes with Intermediate AMD with and without Retinal Pigment Abnormalities

**DOI:** 10.1097/OPX.0000000000001624

**Published:** 2020-12-30

**Authors:** Marilyn E. Schneck, Lori A. Lott, Gunilla Haegerstrom-Portnoy, Susan Hewlett, Bonnie M. Gauer, Ali Zaidi

**Affiliations:** 1 The Smith-Kettlewell Eye Research Institute, San Francisco, California; 2 School of Optometry, University of California, Berkeley, Berkeley, California; 3 Bonnie M. Gauer, OD, MS, LLC, Roseburg, Oregon; 4 Pacific Eye Associates, San Francisco, California

## Abstract

In intermediate AMD, a simple, clinically feasible vision test of sensitivity to radial deformation is significantly more impaired in eyes with hyperpigmentation than in eyes with large drusen but normal retinal pigmentation, consistent with the former's increased risk of progression to advanced AMD. This ongoing longitudinal study will determine whether this vision measure is predictive of progression to advanced AMD.

**PURPOSE:**

This study aimed to determine whether simple, clinically feasible psychophysical measures distinguish between two levels of intermediate AMD that differ in their risk of progression to advanced AMD: eyes with large macular drusen and retinal pigment abnormalities versus eyes with large macular drusen without pigment abnormalities. Abnormal pigmentation in the presence of large drusen is associated with a higher risk of development of advanced AMD.

**METHODS:**

Each eye of 39 individuals with the same form of intermediate AMD in both eyes was tested monocularly on a battery of vision tests. The measures (photopic optotype contrast sensitivity, discrimination of desaturated colors, and sensitivity to radial deformation [shape discrimination hyperacuity]) were compared for both dominant and nondominant eyes. ANOVA with eye (dominant or nondominant) as a within-subject factor and retinal status (pigmentary abnormalities present or absent from the macula) as a between-subject factor was used to determine statistical significance.

**RESULTS:**

Sensitivity to radial deformation was significantly reduced in eyes with large drusen and pigment changes compared with eyes with large drusen and normal retinal pigmentation (−0.40 ± 0.04 vs. −0.61 ± 0.02, respectively; *F* = 13.31, *P* = .001).

**CONCLUSIONS:**

In the presence of large macular drusen, performance on a shape discrimination task is related to the presence versus absence of abnormal retinal pigmentation, being poorer in the higher-risk group, supportive of the measure's potential to predict progression to advanced AMD.

**Figure:**
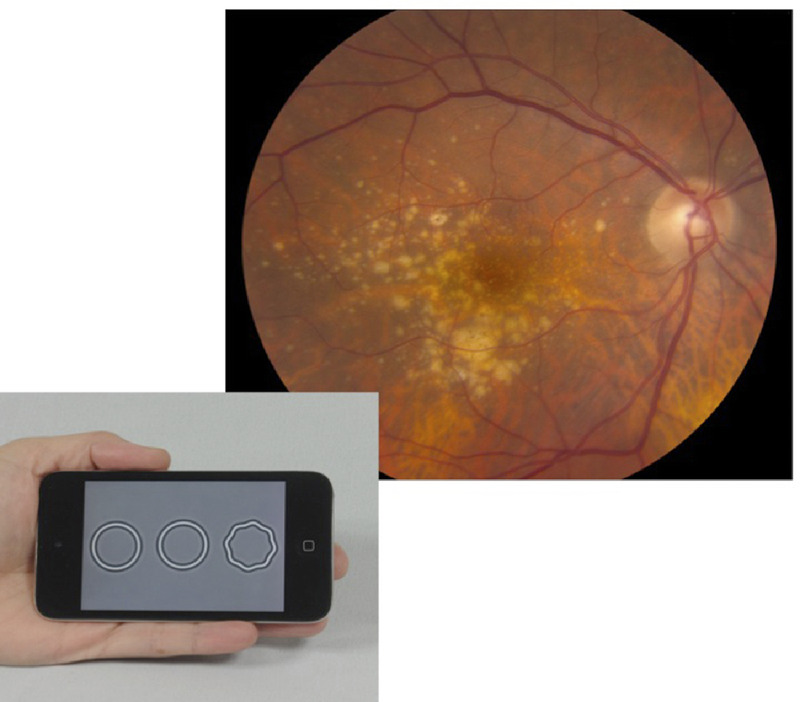


## 

Age-related macular degeneration (AMD) is a progressive, potentially blinding eye disease and is the leading cause of irreversible vision loss among individuals older than 50 years. Wong and colleagues^1^ performed a meta-analysis of worldwide AMD prevalence estimates from 39 cross-sectional studies. The reported global prevalence rates of early and late AMD were 8.01 and 0.37%, respectively, indicating that only a small fraction (~5%) of individuals who develop the early stages of AMD progress to having the advanced forms of the disease, central geographic atrophy or choroidal neovascularization.

As new treatments aimed at earlier, sight-preserving interventions are developed, predicting which individuals with early or intermediate AMD will go on to develop an advanced form of the disease becomes increasingly important for clinical trial design. Identification of individuals with early stages of AMD at great risk of progression is also important for medical management.

A number of genetic, environmental, and demographic risk factors for progression to advanced disease have been identified.^2–5^ Several quantitative models that are predictive of advancement have been developed.^3–5^ Fundus signs, such as the presence of medium or large drusen or the presence of retinal pigmentary abnormalities, are also powerful predictors of the likelihood of progression to advanced disease.^6^

Visual function measures have potential as predictors of progression of AMD from earlier stages to advanced disease.^7–11^ Inclusion of vision measures may strengthen models incorporating other known risk factors.

Ultimately, evaluation of visual function measures as predictors of late AMD requires a longitudinal design with years of follow-up. Cross-sectional studies determining associations between candidate vision measures and known risk factors (such as fundus signs) also have value. A candidate measure may show more frequent or more severe abnormalities in eyes with fundus signs associated with greater risk and more severe disease. We previously found that three measures of visual function each were independently associated with disease severity level in a multinomial generalized estimating equations model.^12^ A shape discrimination hyperacuity task,^13,14^ the Adams desaturated D-15 color vision test,^15,16^ and photopic contrast sensitivity assessed with the MARS chart^17^ were independently associated with the presence of intermediate AMD (characterized by the presence of large drusen with or without pigment changes) when compared with those with a lower risk of advancement, those with no AMD (no drusen or small drusen only), or those with early AMD (defined as medium drusen with no pigment abnormalities).^12^

The changes in visual function may relate to anatomical changes in the retina seen in eyes with genetic risk and signs of intermediate AMD, including increased cone spacing and decreased density.^18^ Also reported is photoreceptor layer thinning over drusen that may be locally associated with drusen, although these findings are not universal.^19–21^ The increased inhomogeneity and cone spacing may lead to undersampling, which could negatively impact visual functions.

Additional changes to the retinal anatomy accompany pigmentary abnormalities. The structural and functional correlates of hyperpigmentation seen on fundus photography have not been fully determined, but spectral domain optical coherence tomography provides insight. The hyperreflective foci overlying drusen in AMD^19^ show significant spatial correspondence with regions of hyperpigmentation on fundus photographs^22–25^ and may reflect the same pathology.^24^ Hyperreflective foci in the outer retina likely reflect disruption and migration of the retinal pigment epithelium into the neurosensory retina,^24,25^ which proceeds from the outer to inner retina over time.^26^ The decrease in cone density, increased inhomogeneity, and intrusion of the retinal pigment epithelium may be expected to particularly impact measures that pool spatial information over an extended retinal area.

Here we compared the performance of two groups of eyes, both with intermediate AMD, which differ in severity and progression risk: eyes with large drusen only and eyes with both large drusen and pigmentary abnormalities. The risk of the group with pigmentary abnormalities developing advanced AMD is more than twice that of the group with large drusen only.^6^ We hypothesized that those with pigmentary abnormalities would have poorer performance on these measures, supporting the assertion that the measures may serve as discriminators of disease severity and predictors of AMD progression.

## METHODS

This research was reviewed and approved by the Smith-Kettlewell Institutional Review Board. Before all procedures, written informed consent was obtained from each participant after an explanation of procedures and after any questions were answered. The study adheres to the tenets of the Declaration of Helsinki.

### Participants

As part of a larger, ongoing longitudinal study, the goal of which is to determine the value of vision measures as predictors of progression to late-stage disease, older individuals were recruited and tested on an array of vision measures and were seen by a collaborating ophthalmologist or optometrist (author BMG) for a complete fundus examination and fundus photography within 2 months of participation. More specifically, 48% of participants had eye examinations before the visual function test session (mean [standard deviation], 36.3 [13.3] days; range, 20 to 60 days), and 52% had eye examinations after the vision testing (mean [standard deviation], 32.1 [20.9] days; range, 0 to 58 days). The data reported here are the initial “baseline” data.

Participants were recruited from several sources: two medical retina practices in San Francisco, CA; an optometry practice in Roseburg, Oregon (author BMG); and our earlier Smith-Kettlewell Institute study of vision in aging.^27^

Eyes were examined for the presence of other ocular diseases (e.g., amblyopia, corneal scarring, glaucoma, and diabetic retinopathy) and were excluded if any disease was present. Prior ocular surgery other than uncomplicated cataract surgery was a criterion for exclusion. Cataract (prior uncomplicated cataract surgery or current cataract) was not an exclusion criterion unless it was associated with acuity worse than 20/40. A visual acuity of 20/40 or better was required for inclusion of an eye in the study. Presence of advanced AMD (choroidal neovascularization or central geographic atrophy) in either eye was an exclusion criterion.

Based on color fundus photography, eyes were classified as having (within two disc diameters of the fovea) no AMD (no or only small drusen and no pigmentary changes), early AMD (medium drusen, >63 and ≤125 μm, and no AMD pigmentary abnormalities), and intermediate AMD (presence of large drusen, >125 μm, with or without AMD pigmentary abnormalities), based on the five-level Beckman Initiative for Macular Research Classification Committee classification system.^6^

Of the 237 eyes that qualified and are included in the larger study, 122 were classified as having intermediate AMD as defined previously. From among these eyes with intermediate disease, individuals with bilateral large macular drusen were identified. Only eyes with the same retinal status in the dominant and nondominant eyes were included, as fellow eye status affects risk. A total of 42 eyes (from 21 individuals) with large drusen only and 36 eyes (from 18 individuals) with large drusen plus pigmentary abnormalities in the macula were identified and included in this report. All eyes with pigmentary abnormalities had hyperpigmentation. The vast majority (93.6%) of the overall sample was White.

### Procedures

Individuals were refracted by an optometrist (authors SH or BMG) and tested with best correction (trial lenses) for the near (40 cm) test distance. Testing was monocular with the eye tested first (right or left) determined by random assignment.

Eye dominance at near was determined using a near hole-in-the-card test based on the method used by Rice et al.^28^

The two groups were compared in terms of age, self-reported years of education, and cognitive status assessed at the time of testing using the Mini-Mental State Examination.^29^ The Mini-Mental State Examination is a brief, standardized test including components assessing orientation, attention, short-term memory, language, and the ability to follow simple instructions. A score <25 indicates the possibility of cognitive impairment and was an exclusion criterion for this study.

### Vision Measures

Best-corrected visual acuity at near was measured for each eye using the high-contrast chart of the SKILL Card^30^ presented at a 40-cm test distance with chart background luminance at 150 cd/m^2^. The subject was required to name letters proceeding down the chart until three of five letters on a line were misidentified. Scoring was letter by letter.

The three measures of interest were part of a larger psychophysical test battery described in more detail elsewhere.^12^

Contrast sensitivity was measured using the MARS charts^17^ presented at a 40-cm test distance with chart background luminance at 150 cd/m^2^. Testing started at high contrast and proceeded to lower contrast down the chart and continued until the participant made two consecutive errors.

Shape discrimination hyperacuity, the ability to detect radial deformation, was measured for radial frequency patterns after the method of Wang et al.^13,14^ Stimuli were presented on an iPod at a 40-cm test distance. Thresholds for sinusoidal deformation from circularity were determined using a three-alternative forced-choice staircase procedure. Participants were given one practice threshold measure, followed by the measurement of two thresholds for each eye. If the two test thresholds differed by ≥0.30 log units, an additional measure was made. The mean of the two (or three) test thresholds was used. Testing time was typically about 2 minutes per eye.

Color vision was assessed using the Adams desaturated D-15 arrangement test under illuminant C.^15^ The Adams version of the test has a saturation of 3, whereas the perhaps more familiar Lanthony desaturated D-15^31^ has a saturation of 2. In addition, the Lanthony test is lighter, with a Munsell value of 8, compared with the Adams version, which has a Munsell value of 5. The color confusion score was calculated,^32^ with a perfect arrangement yielding a color confusion score of 0. Color confusion score represents the distance (as a percentage) in color space traveled by the order of the caps as arranged by the subject compared with that of a perfect order. Thus, a color confusion score of 30 indicates that the distance in color space of the subject's arrangement was 30% larger than that of a perfect arrangement. For those arrangements that produced a color confusion score ≥30, the criterion for failure,^33^ which indicates the presence of a color defect, the angle was calculated to determine defect type (blue/yellow, red/green, or nonselective) using the method of Vingrys and King-Smith.^16^

### Statistical Testing

The demographic characteristics of the two groups of participants (large drusen only vs. large drusen with pigment abnormalities) were compared using two-tailed *t* tests or the χ^2^ test as appropriate.

To test the hypothesis that the presence of pigmentary abnormalities is associated with poorer vision in eyes with large drusen, log contrast sensitivity, shape discrimination thresholds (logMAR), and desaturated D-15 color confusion scores were each compared between groups using ANOVA, with retinal status as a between-subject factor and eye dominance as a within-subject factor.^34^ Color confusion scores were not normally distributed and were therefore log transformed to approximate the normal distribution. There were four instances of a color confusion score of 0 (no errors in cap order) in the group with large drusen only and two in the group with large drusen and pigment abnormalities. These values are not amenable to the log transform and were assigned the log value of 0.8, a value smaller than the smallest color confusion score of a single single-place error. This value was chosen to least inflate the variance of the measure.

### Power Calculations

For both contrast sensitivity and shape discrimination hyperacuity, we expect a clinically significant difference to be at least 0.20 log units. In the baseline sample of participants with intermediate AMD,^12^ the standard deviation for contrast sensitivity was 0.14 log units, and that for shape discrimination hyperacuity was 0.22 log units. The effect sizes (Cohen *d*) for the two variables are 0.20/0.14 = 1.43 and 0.20/0.22 = 0.91, respectively. Given the sample size for the current analyses and assuming α < 0.05, a priori power is expected to be >0.90 for both variables.

For color discrimination, a clinically significant difference should be at least 0.30 log units. The standard deviation for desaturated log color confusion scores for baseline participants in the intermediate AMD group was 0.45 log units. Cohen *d* for color discrimination (0.30/0.45 = 0.67) is a moderate effect size, but assuming α < 0.05, power should still be at least 0.80 for this sample size.

To determine the potential clinical utility of the measure(s) that shows significant differences between the retinal status groups, receiver operating characteristic (ROC) analysis was performed separately for dominant and nondominant eyes, and the area under the curves (AUC) was calculated.

## RESULTS

Table 1 includes demographic data for the two groups and *P* values for the associated *t* test or χ^2^ test. Although the group with large drusen with pigment abnormalities was an average of 4.5 years older than the group with large drusen only, the difference was not significant. The two groups were very similar in terms of years of education and cognitive function as assessed by the Mini-Mental State Examination.^29^ None were cognitively impaired, defined as a Mini-Mental State Examination score <25. Women made up approximately half of each group.

**TABLE 1:** Characteristics of the study participants comprising the group with large drusen only and the group with large drusen with pigment abnormalities

	LDO (n = 21)	LDP (n = 18)	*P***_(two-tailed)_**
Age (y)	73.3 (2.34)	77.6 (2.34)	.21
Percent female	53.5	54.0	.84
Years of education	16.4 (0.58)	17.5 (0.66)	.21
MMSE	28.9 (0.25)	28.2 (0.47)	.19

Values are means with standard errors in parentheses. LDO = eyes having large drusen only in the macula; LDP = eyes having both large drusen and abnormal pigmentation in the macula; MMSE = Mini-Mental State Examination.

**FIGURE 1:**
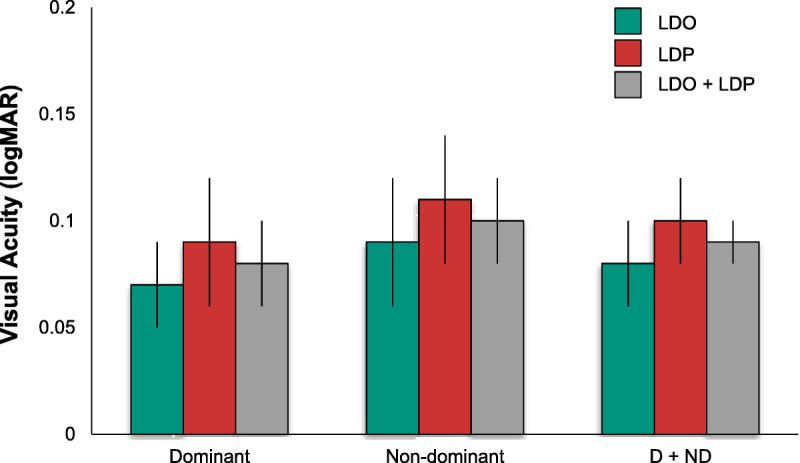
Near visual acuity by retinal status and eye dominance. Mean visual acuity (logMAR) for the dominant (left) and nondominant (center) eyes and combined dominant and nondominant eyes (right; D + ND) with macular large drusen only (LDO) or large drusen and pigment abnormalities (LDP) or the combined groups (LDO + LDP). Error bars are ±1 standard error of the mean. Visual acuity did not vary significantly with eye dominance or retinal status.

Fig. 1 presents the mean visual acuity and standard error of the mean for each subgroup. Differences between dominant (mean, 0.08 ± 0.02) and nondominant (mean, 0.10 ± 0.02) eyes and between the group with large drusen only (mean, 0.08 ± 0.017) and the group with large drusen with pigment abnormalities (mean, 0.10 ± 0.02) were small, equivalent to one letter (0.02 logMAR) on the chart. The ANOVA indicated that these differences were not statistically significant (*F* = 1.29 [*P* = .26] and *F* = 0.35 [*P* = .56], respectively) and that there was no interaction between eye dominance and retinal status (*F* = 0.00, *P* = .96). The small differences reflect the fact that visual acuity was a criterion for inclusion. For all subgroups, the mean visual acuity was about 20/25.

**FIGURE 2:**
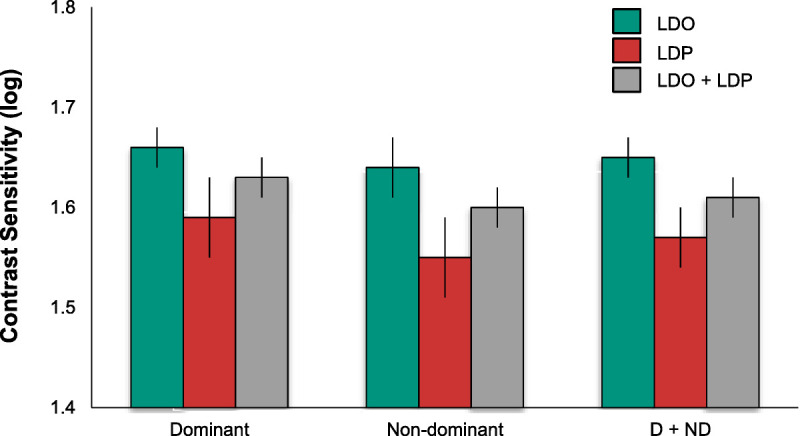
Contrast sensitivity by retinal status and eye dominance. Mean optotype contrast sensitivity (in log units) for the dominant (left) and nondominant (center) eyes and combined dominant and nondominant eyes (right; D + ND) with macular large drusen only (LDO) or large drusen and pigment abnormalities (LDP) or the combined groups (LDO + LDP). Error bars are ±1 standard error of the mean. Contrast sensitivity did not vary significantly with eye dominance or retinal status.

**FIGURE 3:**
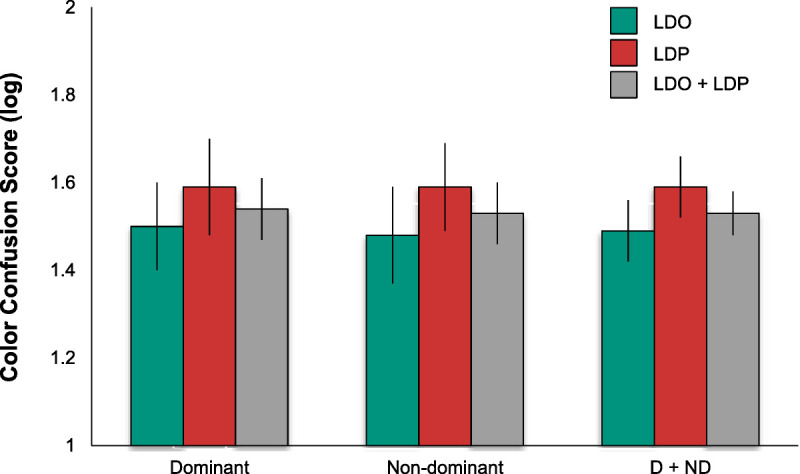
Color discrimination error score (color confusion score; CCS) by retinal status and eye dominance. Mean CCS (log units) for the dominant (left) and nondominant (center) eyes and combined dominant and nondominant eyes (right; D + ND) with macular large drusen only (LDO) or large drusen and pigment abnormalities (LDP) or the combined groups (LDO + LDP). Larger values indicate poorer chromatic discrimination. Error bars are ±1 standard error of the mean. Color discrimination did not vary significantly with eye dominance or retinal status.

**FIGURE 4:**
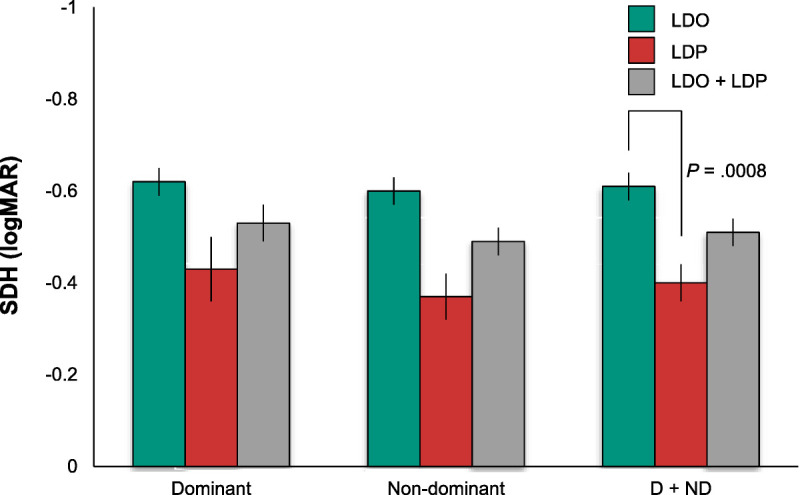
Shape discrimination hyperacuity (SDH) by retinal status and eye dominance. Mean SDH (logMAR) for the dominant (left) and nondominant (center) eyes and combined dominant and nondominant eyes (right; D + ND) with macular large drusen only (LDO) or large drusen and pigment abnormalities (LDP) or the combined groups (LDO + LDP). Note that the ordinate is inverted such that more negative values (better sensitivity) are plotted upward. Error bars are ±1 standard error of the mean. Shape discrimination hyperacuity was significantly less acute in LDP than LDO eyes.

The mean scores and associated error bars (standard error of the mean) for the vision measures of interest (contrast sensitivity, desaturated D-15 color confusion score, and shape discrimination hyperacuity) are plotted in Figs. 2 to 4, respectively, for the two groups. Note that larger values indicate better (log) contrast sensitivity, but lower log color confusion score values reflect better color discrimination. Lower (more negative) values indicate better shape discrimination hyperacuity thresholds, which are in logMAR units. Also note that the shape discrimination hyperacuity data in Fig. 4 are plotted with an inverted ordinate, with values becoming more negative (more sensitive) going up. Finally, note that ordinate scales are not comparable in range across figures.

Although contrast sensitivity is somewhat higher in the eyes with large drusen only than in the eyes that also have abnormal retinal pigment for both dominant and nondominant eyes (Fig. 2), contrast sensitivity did not differ significantly between the eyes with large drusen only (mean, 1.65 ± 0.02) and the eyes with abnormal pigment in addition to large drusen (mean, 1.57 ± 0.03), although the 0.08-log-unit difference in means approached significance (*F* = 3.73, *P* = .06). Contrast sensitivity did not differ between dominant and nondominant eyes (means, 1.62 ± 0.02 and 1.60 ± 0.02, respectively; *F* = 2.36, *P* = .13). There was no interaction between eye dominance and retinal status (*F* = 0.38, *P* = .54).

Similarly, although the mean color confusion scores of the eyes with retinal pigment abnormalities (mean, 1.59 ± 0.07) are slightly higher (indicating poorer performance) than those of the group with large drusen only (mean, 1.49 ± 0.07), log color confusion score (Fig. 3) showed no significant effects of retinal status (*F* = 0.69, *P* = .41). There was no effect of eye dominance (means, 1.54 ± 0.07 and 1.53 ± 0.11 for dominant and nondominant eyes, respectively; *F* = 0.02, *P* = .92). The interaction between eye dominance and retinal status was also not significant (*F* = 0.03, *P* = .88).

Shape discrimination hyperacuity thresholds (Fig. 4) also showed no significant effect of eye dominance (means, −0.53 ± 0.04 and −0.49 ± 0.03 for dominant and nondominant eyes, respectively; *F* = 3.03, *P* = .09) and no significant retinal status by eye dominance interaction (*F* = 0.50, *P* = .48). However, shape discrimination thresholds showed a significant effect of retinal status. Mean threshold was higher (mean sensitivity was lower) in the eyes with abnormal pigment compared with the eyes with large drusen only (−0.40 ± 0.04 vs. −0.61 ± 0.02, respectively; *F* = 13.31, *P* = .001).

Comparison of the standard errors of the three measures indicates that the variability of the color vision data is considerably greater than that for the other two measures. Senescent lens changes are known to impact color vision, resulting in blue/yellow type errors.^35^ Cataracts were present in some individuals of each retinal status group, whereas others had no cataract (the four youngest members of each retinal status group) or were pseudophakic after cataract extraction (Table 2).

**TABLE 2:** Age and vision measures by cataract status for the group with large drusen only and the group with large drusen and pigment abnormalities

Cataract status	Large drusen only					Large drusen and pigment				
	n	Age (y)	CS	SDH	DesatCCS	n	Age (y)	CS	SDH	DesatCCS
No cataract	4	55.37 (0.42)	1.66 (0.13)	−0.73 (0.06)	1.11 (0.39)	4	59.36 (2.04)	1.74 (0.08)	−0.41 (0.50)	1.32 (0.50)
Any current cataract	28	74.43 (8.62)	1.64 (0.12)	−0.59 (0.16)	1.67 (0.39)	12	78.02 (7.48)	1.52 (0.19)	−0.42 (0.28)	1.72 (0.45)
Cataract surgery	10	83.07 (6.10)	1.68 (0.08)	−0.61 (0.07)	1.11 (0.31)	20	80.99 (7.85)	1.57 (0.12)	−0.38 (0.15)	1.57 (0.41)

Values are means with standard deviation in parentheses. CS = contrast sensitivity (log); DesatCCS = desaturated color confusion score (log); SDH = shape discrimination hyperacuity (logMAR).

For this (noninferential) exploration, dominant and nondominant eyes were combined within each group. For the group with large drusen only, the no-cataract group and cataract surgery group had similar performance (mean log color confusion score, 1.11), which was 0.56 log units better than the eyes with current cataract. The presence of cataract is associated with poorer color discrimination in this group. In the eyes with pigmentary abnormalities in addition to large drusen, the mean log color confusion score of the eyes with any cataract of 1.72 is somewhat higher than that of the pseudophakic eyes (1.57). The younger no-cataract group had a better color discrimination with a mean log score of 1.32. Thus, cataract status impacted color vision test scores and may have contributed to the large variability observed in that measure. However, note that even within a cataract status group, the standard deviation is larger than that of the other two visual function measures.

All eyes with cataract in both the retinal status groups that failed the color vision test had a blue/yellow error pattern, as did 50% of the noncataractous eyes in the group with large drusen only and 92.3% of those with pigmentary changes. The remainder had nonselective defects.

The large effect of cataract on color vision test results may limit its usefulness in older populations. We examined the impact of cataract status on the other two vision measures as well, to see whether they were similarly affected.

The other two measures show less effect of cataract on mean scores. The contrast sensitivity differences between the eyes with cataract and the pseudophakic eyes were small, 0.04 log units (2%) for the eyes with large drusen only and 0.05 log units (3.2%) for the eyes with pigment changes, with sensitivity slightly higher in the pseudophakic eyes.

The mean shape discrimination hyperacuity score for the eyes with large drusen only with cataract (−0.59) is very similar to that of the pseudophakic eyes (−0.61), a 3.3% difference. The difference was 10% for the eyes with pigmentary changes (−0.42 vs. −0.38 for the eyes with cataract and pseudophakia, respectively).

**FIGURE 5:**
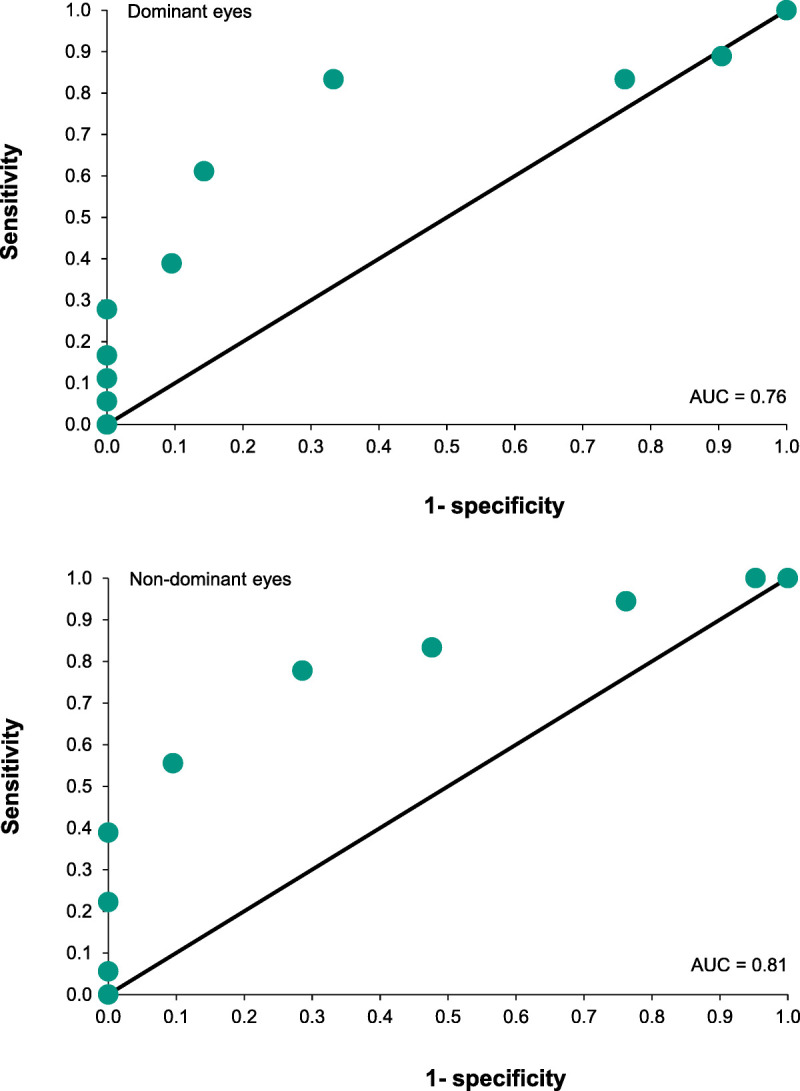
Receiver operating characteristic curves for the shape discrimination hyperacuity scores for the dominant (upper) and nondominant (lower) eyes for the two retinal status groups (large drusen only vs. large drusen with pigment abnormalities). The diagonal line represents 0 discriminability, or chance performance. The area under the curve (AUC), an index of accuracy, is given in each graph.

To determine whether the shape discrimination hyperacuity test has clinical utility for determining retinal status, ROC analysis was carried out for this measure, separately for the dominant and nondominant eyes. The AUC provides an index of the degree to which it distinguishes the two retinal status groups. The results are shown in Fig. 5. The discrimination between groups was fair for the dominant eyes (AUC, 0.76; sensitivity, 83.3; specificity, 76.2) and good for the nondominant eyes (AUC, 0.81; sensitivity, 77.8; specificity, 71.4).

## DISCUSSION

We examined visual function in two groups of eyes with intermediate AMD, one with large drusen as the only fundus sign and one with hyperpigmentation in addition to large drusen. The risk of progression of the latter group is greater than that of the eyes with large drusen only. Visual acuity did not differ between the group with large drusen only and the group with large drusen with retinal hyperpigmentation, nor did color discrimination or contrast sensitivity, although this difference for contrast sensitivity approached significance. We find that one measure, shape discrimination hyperacuity,^13,14^ is significantly poorer in the group with pigmentary abnormalities, supporting the potential of the measure to distinguish between disease severity levels and potentially aid in the prediction of progression to advanced disease. ROC analysis indicated that the measure had fair to good discrimination of eyes with large drusen and pigment from eyes with large drusen only.

Dimitrov and colleagues^36^ compared visual function in eyes with soft drusen with eyes with both soft drusen and pigmentary abnormalities and reported that hypopigmentation, not hyperpigmentation, was associated with poorer function on some measures (short-wavelength cone thresholds and sensitivity to 14-Hz flicker). Wang et al.^13^ reported that eyes with drusen and hyperpigmentation tended to have somewhat (0.15 log units) poorer shape discrimination hyperacuity compared with eyes with drusen alone for large (mean radius of 2.0 or 2.5°) targets, but this difference was not statistically significant perhaps because of the small sample size.^13^ No difference between these groups was found for smaller (1° mean radius) targets like those used here. In contrast, here sensitivity to radial deformation was found to be lower in the presence of retinal hyperpigmentation.

Optimal performance of the shape discrimination hyperacuity task likely involves a global shape mechanism that pools spatial information over an extended retinal area.^14,37,38^ We previously reported decreased function in eyes with intermediate AMD compared with eyes with early or no macular degeneration.^12^ This decline in function may be related to the inhomogeneity of the macula that is characteristic of intermediate AMD, as described in the Introduction.^18–26^ Increased photoreceptor spacing in patches of the retina may lead to undersampling of the image and reliance on only a portion of the radial pattern for making the judgment, which is expected to reduce performance.^13,14^

Pigmentary changes are associated with poorer shape discrimination hyperacuity performance than that observed in intermediate age-related macular generation with large drusen and normal pigment. The poorer performance in the presence of pigmentary abnormalities may relate to the alterations in the retina that occur in the presence of hyperpigmentation, as described in the Introduction. The migration of the retinal pigment epithelium into the sensory retina^24,25^ is likely to further disrupt both the regularity and orientation over that seen in the presence of drusen and thus the function of the photoreceptor mosaic. Such changes may contribute to the poorer performance in the group with hyperpigmentation of the retina in addition to large drusen.

Like shape discrimination hyperacuity, color discrimination is impaired in intermediate AMD compared with age-similar eyes with no AMD or early AMD.^12^ However, the 0.14-log-unit difference in mean color confusion score between the two groups observed here did not reach statistical significance perhaps because of the great variation (a nearly 2-log-unit range of values) among the color confusion scores in each group, reflected in the large standard errors (Fig. 3). Variations in cataract status among the eyes in each group may have contributed to the large variability; eyes with cataract tended to have higher error scores compared with pseudophakic or noncataractous eyes, particularly in the absence of pigmentary abnormalities. The dependence of color vision performance on cataract status potentially limits its clinical value because interpretation of the test scores requires knowledge of cataract status.

Photopic contrast sensitivity did not differ significantly between the group with large drusen only and the group with large drusen with retinal pigment abnormalities. Furthermore, comparison of the contrast sensitivity of the AMD groups with a group with no AMD (data not shown) indicated that the mean contrast sensitivity of the group with large drusen only was not much reduced compared with that of those without the eye disease (mean differences of 0.05 and 0.08 log units for the dominant and nondominant eyes, respectively). This result suggests that the significant difference in contrast sensitivity between eyes with no AMD and eyes with intermediate AMD recently reported by Lott and colleagues^12^ may be attributed to the decreased performance of those eyes with pigmentary changes in their intermediate group. Those authors did not separate intermediate eyes based on the presence or absence of pigmentary abnormalities.

A limitation of the study is that, in light of the sample size, cataract status was not taken into account in our ANOVA and could have affected the results by inflating variability, particularly with respect to the desaturated D-15 scores.

Larger longitudinal studies are also needed to determine whether the addition of this visual function measure strengthens the predictive models of progression to late-stage AMD that incorporate other known risk factors, such as fundus appearance and genetic risk. In addition, studies including eyes of non-Whites are needed. Nearly all (93.6%) of the overall study sample was White, and the generalizability to other groups is not known.

In summary, performance on the shape discrimination hyperacuity test is associated with and discriminates fairly well between retinal signs that are associated with different risks of progression to advanced AMD. The test thus may have promise as a predictor of the development of choroidal neovascularization or central geographic atrophy. The test requires minimal equipment (a smartphone) and takes just a few minutes to perform and thus recommends itself as a tool to aid in the determination of the presence of pigmentary changes, thus increasing the risk of progression to advanced disease, in eyes with large drusen. The ongoing longitudinal study will determine whether the shape discrimination measure is in fact predictive of advancement to one of the advanced forms of AMD. If this measure were predictive of progression to advanced AMD, the clinical significance would be far-reaching, aiding both the design of clinical trials and patient management.
